# Exploring the future adult vaccine landscape—crowded schedules and new dynamics

**DOI:** 10.1038/s41541-024-00809-z

**Published:** 2024-02-09

**Authors:** Charles H. Jones, Matthew P. Jenkins, B. Adam Williams, Verna L. Welch, Jane M. True

**Affiliations:** Pfizer Inc, 66 Hudson Boulevard, New York, NY 10001 USA

**Keywords:** RNA vaccines, Infectious diseases

## Abstract

Amidst the backdrop of the COVID-19 pandemic, vaccine innovation has garnered significant attention, but this field was already on the cusp of a groundbreaking renaissance. Propelling these advancements are scientific and technological breakthroughs, alongside a growing understanding of the societal and economic boons vaccines offer, particularly for non-pediatric populations like adults and the immunocompromised. In a departure from previous decades where vaccine launches could be seamlessly integrated into existing processes, we anticipate potentially than 100 novel, risk-adjusted product launches over the next 10 years in the adult vaccine market, primarily addressing new indications. However, this segment is infamous for its challenges: low uptake, funding shortfalls, and operational hurdles linked to delivery and administration. To unlock the societal benefits of this burgeoning expansion, we need to adopt a fresh perspective to steer through the dynamics sparked by the rapid growth of the global adult vaccine market. This article aims to provide that fresh perspective, offering a detailed analysis of the anticipated number of adult vaccine approvals by category and exploring how our understanding of barriers to adult vaccine uptake might evolve. We incorporated pertinent insights from external stakeholder interviews, spotlighting shifting preferences, perceptions, priorities, and decision-making criteria. Consequently, this article aspires to serve as a pivotal starting point for industry participants, equipping them with the knowledge to skillfully navigate the anticipated surge in both volume and complexity.

## Introduction

As the adult vaccine landscape rapidly evolves, we find ourselves at a crossroad where addressing the status quo of immunization efforts is no longer an option but a necessity. The COVID-19 pandemic served as a stark wake-up call, shattering the comforting illusion of “there is always next year”—a sentiment that echoes the preludes to the 2008 financial crash. This global health crisis exposed the fragmented nature of our adult vaccine infrastructure on both domestic and international fronts, revealing a system that is wholly unprepared for the impending rapid growth in the adult vaccine market. Leaders within the pharmaceutical industry, however, view the challenges within the adult vaccine industry as catalysts for transformation—a chance to reshape the adult vaccine landscape and contribute to a modern-day renaissance that promises improved immunization outcomes in the years to come.

The ongoing and accelerating transformation in adult vaccines is expected to be propelled by the rise of RNA technology, thrusting us into a new era of digital vaccines^1^. Unlike their traditional biologic counterparts, RNA-based solutions are not constrained by the same production process for varying antigens sequences^1^. Instead, the manufacturing process remains largely the same, with only variations in the antigen sequence encoded in the RNA vaccine. This opens the door to conceptualize innovative vaccine designs using a single manufacturing process—a departure from the conventional wisdom that “the process is the product,” and a giant leap forward for future vaccine design and development.

**Fig. 1:**
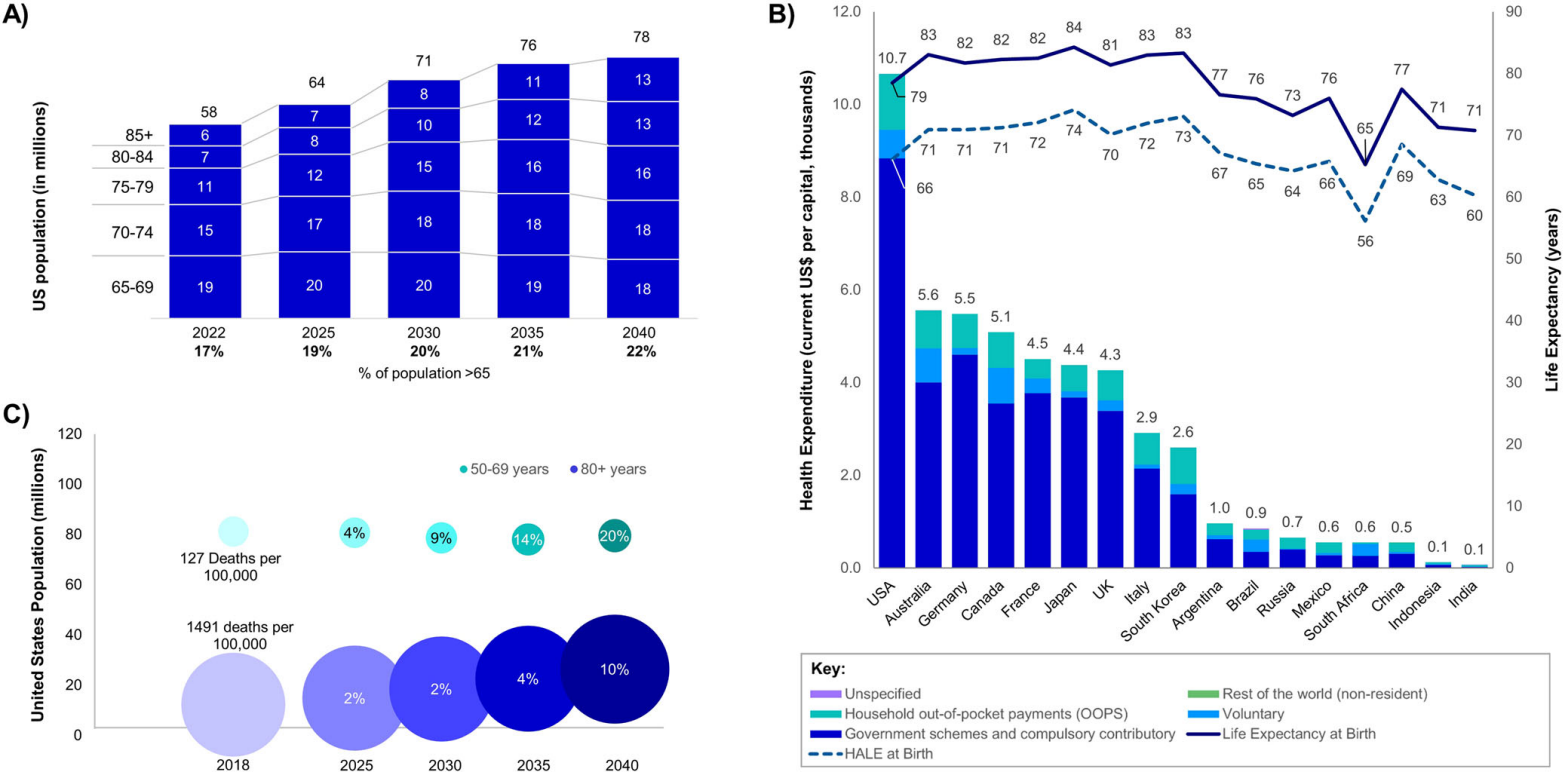
Demographic trends, projected disease burden, and primary healthcare funding in relation to life expectancy. **A** Depicts the growth of the U.S. population segment aged 65 years old and above from 2022 to 2040. Data for 2022 was sourced from the U.S. Census Bureau’s annual estimates (U.S. Census Bureau, Population Division, June 2023), while future projections were obtained from the IHME’s Global Fertility, Mortality, Migration, and Population Forecasts 2017–2100 (IHME, 2020). **B** Highlights the relationship between primary health care funding schemes in select G20 countries (excluding Saudi Arabia and Turkey due to lack of data) and life expectancy at birth and HALE at birth. Health expenditure data was sourced from WHO’s Global Health Expenditure Database and Life expectancy data was retrieved from the Global Health Observatory Data Repository for 2019. **C** Illustrates the projected disease burden for age groups 50–69 years and 80+ years, with bubble size denoting death per 100,000. Percentages correspond to an increase in area. Data is derived from the Global Burden of Disease Study 2016 (IHME, 2018). Note: The age group 70–79 is not represented due to unavailability of data.

The timing of this vaccine revolution is critical. As the world’s population ages, the call for more potent vaccines to safeguard health and wellness rises (Fig. 1A). Currently in the United States, the healthy life expectancy (HALE) is only 66 years, despite it spending more than any other G20 nation on health care (Fig. 1B). However, the older demographics are increasingly embracing self-care and healthy aging, thereby fueling demand for healthcare products that promote longevity, such as vaccines against infectious diseases that pose significant risk to these populations^2^. This need is further driven by the impact infectious diseases have on the economy. Vaccine-preventable diseases (VPDs) account for an estimated 8 to 10 million disease cases in the U.S. alone, resulting in up to $34.9 billion in annual societal costs^3^. However, current vaccination rates in the U.S. for most of these diseases fall significantly short of the Healthy People 2030 targets^4^, even as the burden of infectious diseases is poised to escalate (Fig. 1C). Increasing vaccination rates to achieve these targets could result, over the course of 30 years, in an additional 33 million averted disease cases, a saving of $96 billion in costs, and nearly $83 billion in incremental vaccination costs^5^. The impact of VPDs also goes beyond measurable economic impacts as older adults play an invaluable role in the informal economy, offering childcare, and financial and emotional support^6^. Such contributions cannot be quantified through economic analyses alone.

Technical challenges associated with developing adult vaccines add another layer of complexity and can pose significant barriers. Future vaccines will need to be tailored to different risk groups for optimal efficacy, as immune responses vary across adult populations^7^. This is especially true for older adults, who often exhibit weaker immune responses due to immunosenescence^8^. Therefore, vaccines for older adults may require higher doses or specially designed adjuvants to compensate for this^9^. Diseases prevalent across various age groups could be driven by different strains of the same pathogen, leading to a balancing act in vaccine design. Pneumococcal disease perfectly illustrates this point where the most significant serotypes vary between different age groups^10^. As a result, vaccine makers might prefer to include those serotypes that are most relevant to pediatric populations. This approach was demonstrated in the Shared Clinical Decision-Making (SCDM) decision in 2019, where the protection from pediatric vaccination was considered sufficient for at-risk adults^11^. These complexities have driven prioritization of certain age groups, primarily shifting focus to younger populations due to high vaccine efficacy, lower social contact, and widespread adherence to nonpharmaceutical interventions^12^.

To better understand the evolving adult vaccine market, we conducted a market research study, using the U.S. as a pivotal case study. Through collaboration with key stakeholders from various sectors, we established a foundational understanding of the current and future landscape that will pave the way for subsequent assessments specific to countries or regions. Our research reveals crucial insights into the challenges and weakness of the adult vaccine market which, if not addressed, may quickly become overwhelmed in the face of an evolving and expanding industry. Equipped with this knowledge, we aim to change “there is always next year” from a complacent phrase to an urgent call for innovation, fostering a healthier future with enhanced access, affordability, and awareness for adult vaccines.

## Impending adult vaccine market evolution and growth

Historically, pediatric vaccines have been prioritized over vaccines designed specifically for adults. In fact, the first vaccine approved following the formation of U.S. Food and Drug Administration (FDA) was the diphtheria pediatric vaccine in the early 1920’s, nearly 20 years before the approval of the first adult vaccine for influenza in 1945^13^. This bias was justified in the mid-twentieth century, which saw a surge in the adolescent population due to improved healthcare and the baby-boom following World War II^14^. The vulnerability of this population to common infectious diseases, such as measles, mumps, and rubella, necessitated a robust pediatric immunization program. Other factors, such as the homogeneity of the pediatric population and the frequency at which they interact with the healthcare system^15^, have all led to the development of a strong pediatric immunization program in the U.S. today, consisting of clear guidelines, well-defined immunization schedules, and school entry requirements. Together, these factors have contributed to a vaccination rate among school-aged children of 90%^15^. In contrast, adult vaccines often face challenges such as limited awareness, accessibility, affordability, and vaccine hesitancy, which has resulted in vaccination rates that range from 20 to 62%^16^.

Despite these challenges, the adult vaccine market is experiencing rapid expansion, with a diverse range of anticipated products targeting diseases such as influenza, pneumococcal disease, herpes zoster (Zoster), hepatitis, and human papillomavirus (HPV) (Supplementary Fig. 1). This market, excluding pediatric specific vaccines, was valued at $19.48 billion in 2022 and is projected to reach $27.65 billion by 2028, reflecting a compound annual growth rate (CAGR) of 6.1%^17^. Its growth trajectory is fueled by the increasing prevalence of VPDs in adults, technological advancements like RNA vaccines, and a heightened focus on preventative healthcare.

Over the next decade, we anticipate a tripling in the number of approved vaccine products globally. Today, there are 35 products available for 13 disease areas. Over the next 10 years, we foresee between 100-120 risk-adjusted products (risk-adjusted using probability of technical and regulatory success [PTRS] values by stage of development) designed to protect against 40 different disease areas (Supplementary Fig. 1). Currently, our arsenal of vaccines is primarily divided into two main disease categories: those targeting well-known diseases like influenza, pneumonia, shingles, and COVID-19, and those designed for travel or endemic diseases like hepatitis B, Ebola yellow fever, tick-borne encephalitis, and Japanese Encephalitis. Looking towards the future, we can expect these categories to expand to include nosocomial vaccines, targeting infections acquired in healthcare settings like *Clostridioides difficile* (*C. diff*) and *Staphylococcus aureus* (Staph A). Furthermore, vaccines for diseases with high unmet needs, such as human immunodeficiency virus (HIV), may enter the adult vaccine landscape over the next 10 years.

In addition to expanded vaccine offerings, we also anticipate growing competition, with four historical leaders in the field (GlaxoSmithKline, Merck, Pfizer, and Sanofi) making significant advancements. However, it should be noted that the COVID-19 pandemic has likely reshuffled the adult vaccine landscape^18^, which has become one of the most competitive spaces in pharma. There has been increased pressure from both new entrants with differentiated technology platforms (such as Moderna’s mRNA portfolio and Dynavax’s adjuvant offering) and low-margin, high-volume global players (such as Serum Institute of India, Bharat Biotech, and Sinovac)^18^. Additionally, as best-in-class products likely will not be enough to capture the market, manufacturers are expected to distinguish themselves through differentiated portfolio offerings, rather than individual products^18^.

With the increasing number of adult vaccines entering the market, adult vaccine schedules are expected to undergo substantial expansion over the next decade (Supplementary Fig. 2). The current Advisory Committee on Immunization Practices (ACIP) adult vaccine schedule recommends that individuals aged 18 and above receive vaccines for influenza and COVID-19. It is also recommended that adults aged 50 and above get the Zoster vaccine, while those aged 65 and above are recommended pneumococcal vaccines. Moreover, both vaccines are recommended for adults aged 18 and older who have compromised immune systems. Those under 65 are recommended to get vaccinated against hepatitis B. Recently, two new RSV vaccines have been approved and may be considered for adults aged 60 and older through the process of shared clinical decision-making, as per SCDM guidelines. Looking forward to the next 10 years, we foresee most new vaccines being specifically developed for certain high-risk groups, irrespective of their age. For example, *C. diff* vaccines may be recommended for those aged 65 and above that are admitted to a hospital or long-term care facilities. We anticipate that COVID-19 and RSV vaccines will begin to mirror the pattern seen with the influenza vaccine, possibly evolving into seasonal vaccines^19–21^. This suggests that adults may be recommended to receive these vaccines annually, typically within a three-month window, assuming current seasonal vaccination behavior (agnostic of potential recommendation).

The possibility of a surge in seasonal vaccines could set the stage for a scenario where other, non-seasonal vaccines might find themselves being administered over a compressed three-month period. This potential phenomenon, termed forced seasonality, could pave the way for a convergence of campaigns for new vaccines with those of seasonal vaccines, instigated by the necessity to co-administer them to boost uptake. Such an alignment could pose substantial challenges for immunizers and vaccination delivery sites as they grapple with the task of accommodating new patients and vaccines within a more limited timeframe.

**Fig. 2:**
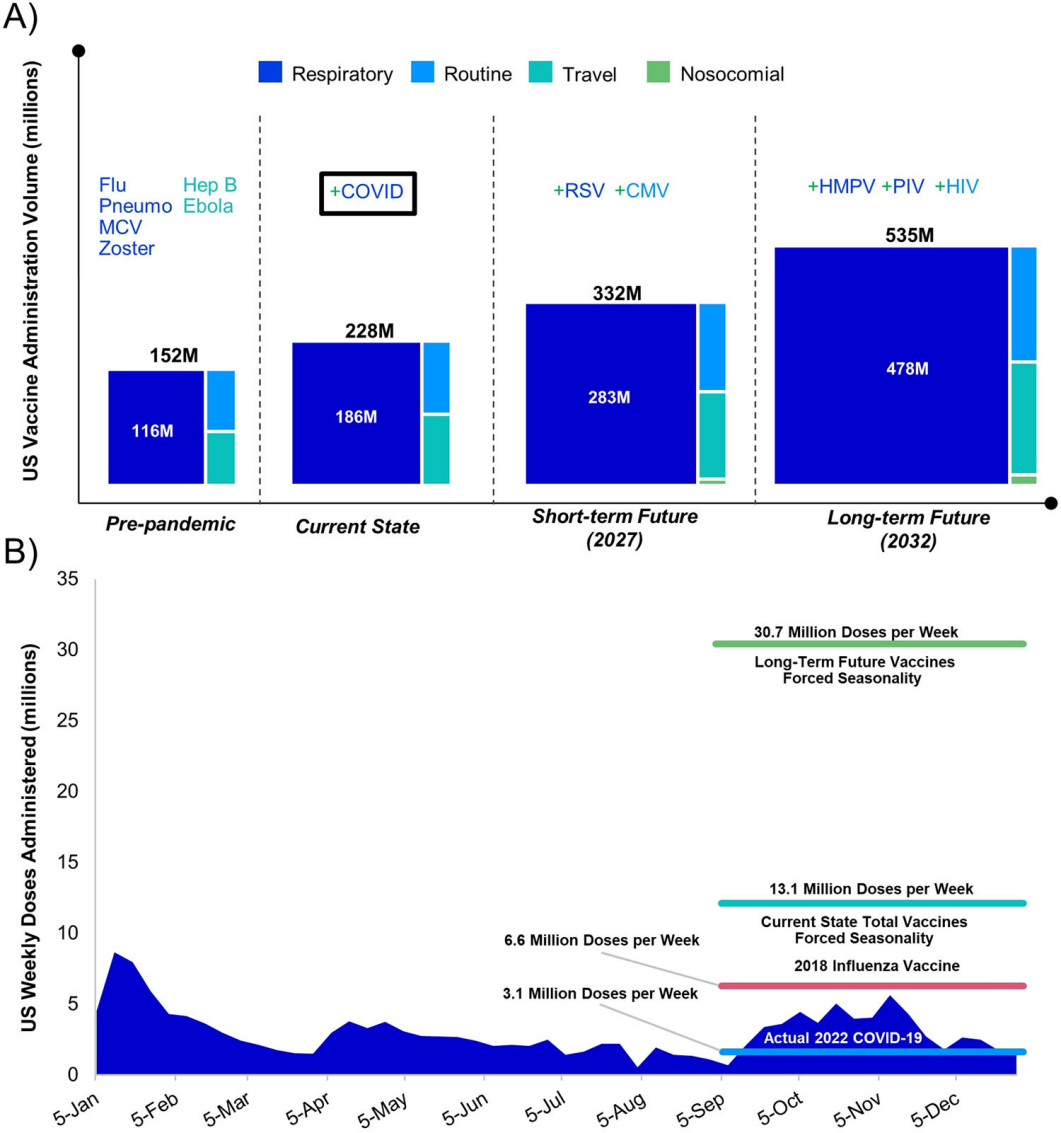
The rising tide of adult vaccination in the United States. **A** Assessment of the projected future vaccine volume expansion in the U.S., by vaccine category, compared with current and pre-pandemic levels. Notable recent and anticipated vaccine launches are called out. The box around COVID indicates that this launch has already occurred. A more comprehensive representation of anticipated vaccine launches can be found in Supplementary Fig. 1. **B** Weekly COVID-19 Vaccine Administrations and Productivity in 2022 and Projections for 2032. This figure presents the number of COVID-19 vaccine doses administered weekly throughout 2022. This data was obtained from the CDC’s COVID Data Tracker. Weekly productivity, represented as millions of doses administered per week, during the peak vaccination season (September to December) for actual 2022 COVID-19 vaccine administrations and 2018 influenza administration is shown. Data for influenza vaccines was obtained from the CDC’s FluVaxView. Comparative data is shown for the total vaccine administration volume in 2022 and projections for total vaccine administration volume in 2032, under an assumed forced seasonality and current vaccination behavior.

While forced seasonality has not yet burdened the immunization infrastructure, it may in the future as the adult vaccine market expands. In fact, if current vaccination rates are held constant, the total annual volume for adult vaccines will surpass 500 million doses dispensed in the U.S. alone by 2032 (Fig. 2A). This estimate accounts for the risk-adjusted products expected to enter the market in the next 10 years, as well as their dosing regimens. Should this occur, this would be a considerable leap from the current 200 million doses administered annually. To put this into perspective, the global influenza vaccine market stands at approximately 600 million doses per year^22^. Assuming forced seasonality as well as current vaccination trends and behaviors in the U.S., we will find ourselves needing to process a volume equivalent to the entire global influenza vaccine market within the U.S. in a three-month span.

This would represent a formidable challenge when compared with the current immunization productivity in the U.S. During the 2022 to 2023 influenza vaccine season, 76% of the season’s total 173.37 million vaccines were administered during the peak season from September to December, according to the Center for Disease Control and Prevention’s (CDC’s) Flu VaxView^23^. In fact, according to the same database, approximately 85% of people 65 and older who were vaccinated by the end of the 2021–2022 season had received their influenza vaccine by the end of November 2021^23^. Averaging out the number of doses administered to that population over the number of days within an influenza vaccine season, this amounted to 1.1 million doses administered each day during that time period. Even with today’s vaccine volume of 200 million doses, assuming forced seasonality from September to the end of the year would result in a productivity of 1.9 million doses per day (13.1 million doses per week). That is significantly higher than the 0.4 million doses per day (3.1 million doses per week) administered during the COVID-19 vaccine’s peak season (Fig. 2B). By 2032, assuming forced seasonality and current vaccination behavior, this could potentially escalate to 4.4 million doses per day (30.7 million doses per week). To cater to the projected annual volume of over 500 million vaccine doses by 2032, we would have to significantly augment this daily productivity rate, which would necessitate not just a change, but a necessitate a major paradigm-shift in our approach to vaccine administration and consumer vaccination behavior.

One solution could be to shift the administration window so that peak vaccination season begins earlier in the year. However, accommodating the growing number of vaccination options would require changes to our current approach. These changes could include expanding access to vaccination services, increasing public awareness and education, and implementing targeted interventions to drive behavior change. To-date, no comprehensive assessment has been conducted on what these changes might be or on stakeholder readiness, leaving many unprepared or even unaware of the evolving adult vaccine market. This presents a significant challenge, as without a clear understanding of the necessary changes and the readiness of stakeholders to implement them, it will be difficult to successfully execute this solution and achieve our productivity goals.

## Breaking down barriers: a study on navigating the challenges of an evolving industry

To better understand the potential challenges and reactions to future states of the adult vaccine landscape, we undertook a market research study comprising both qualitative and quantitative approaches (see Supplementary Information for more study details; Supplementary Fig. 3). Our research methodology was built on an iterative, sequential primary market research design using mixed methods. This encompassed a five-step process that began with fact gathering and hypothesis identification. The initial fact-gathering stage involved an extensive exploration of existing knowledge, data, and available information on the potential evolution of the adult vaccine landscape. Based on this information, we developed hypotheses about potential future market state scenarios.

Qualitative interviews were conducted with key industry stakeholders (i.e., recommenders and funders, stocking and purchasing representatives, immunizers and advocacy groups, and consumers) from the U.S., with the aim of exploring their practices concerning the recommendation, funding, stocking, purchasing, immunizing, and advocacy for vaccines. The stakeholders were provided with the expected changes within the adult vaccine market (Supplementary Fig. 4, Supplementary Fig. 5, Supplementary Fig. 6, and Supplementary Fig. 7). Two future scenarios were then presented to each stakeholder (Supplementary Fig. 8). The first scenario was optimistic, showing a future where stakeholders adapt and seize new opportunities, while the second scenario was pessimistic, illustrating a future where stakeholders maintain their usual practices that might not fit the evolving market. In this way, we sought to assess the stakeholders for their awareness of changes within the market and their perspectives on how these changes might be handled in the future.

We also conducted quantitative surveys among immunizers and adult consumers to measure the impact and compromises linked with alternative scenarios within the adult vaccine landscape. Our analyses aimed to characterize the degree of impact and trade-offs concerning U.S. future state scenarios.

While the totality of results from this study is too extensive for this perspective, we will center our focus on the crucial dynamics and issues that demand attention, ensuring stakeholders within the adult vaccine market are better informed as to how handle increasing demand without the system becoming overwhelmed and collapsing.

### Ignorance, finger pointing, and overconfidence

**Fig. 3:**
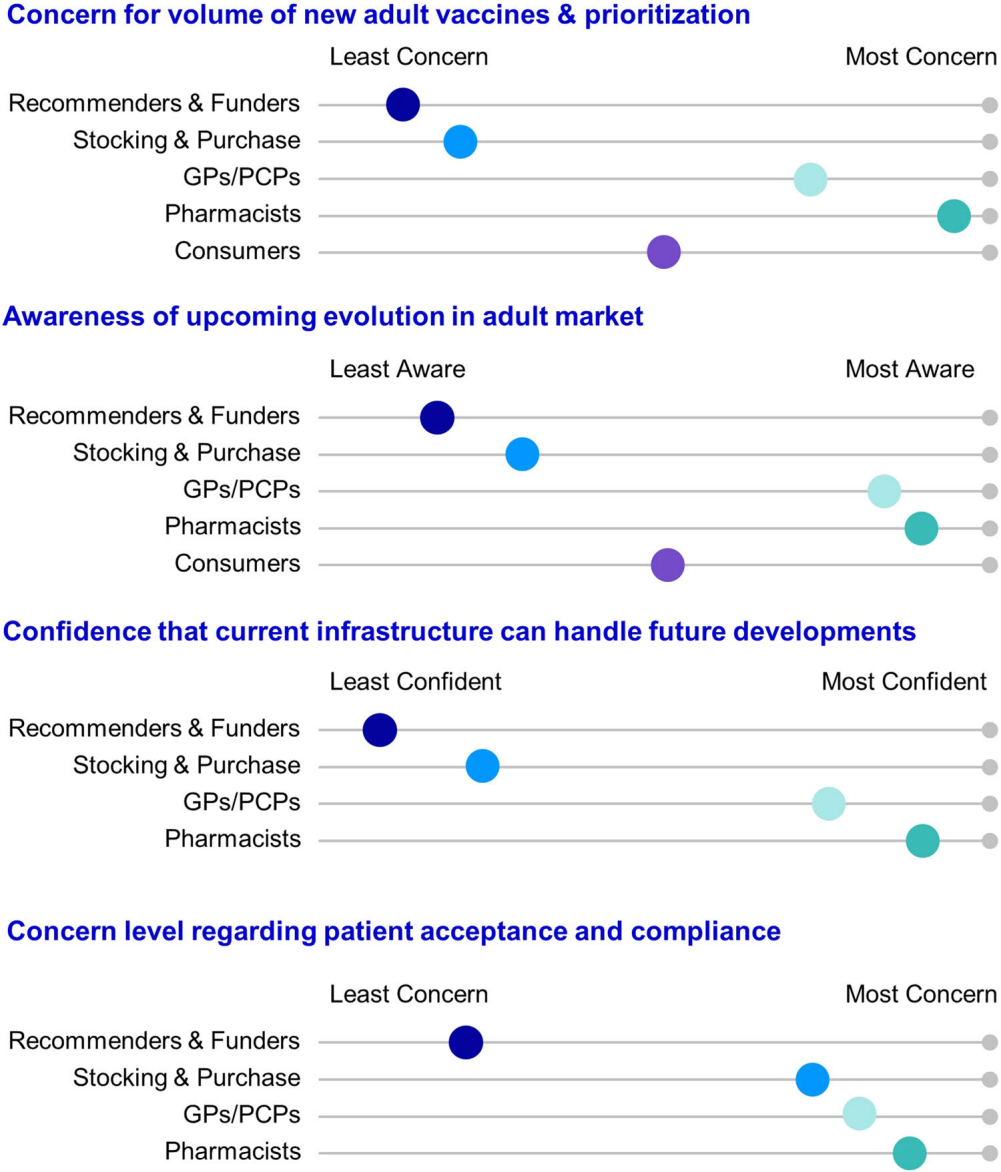
Stakeholder preferences across sensitivity scenarios. Visual comparison of stakeholder preferences is provided across sensitivity scenarios. The sensitivity levels were determined using insights gained through the assessment of qualitative statements gathered from stakeholders (Recommenders & Funders, Stocking & Purchase, General Practitioners [GPs] & Primary Care Physicians [PCPs], Pharmacists, and Consumers). These quantitative statements were gathered in response to future market scenarios.

Through interviewing and surveying key adult vaccine market stakeholders, we found that many stakeholders may not be fully cognizant of the impending wave of adult vaccines. The primary reason for this ignorance was the absence of incentives to assess situations beyond the current fiscal year, which fostered short-term thinking and hindered the identification of potential long-term effects within the adult vaccine market. This lack of awareness can also potentially lead to a lack of preparation and resulting missed opportunities as the stakeholders at the top of the value chain underestimate the challenge to adopt future adult schedules. Immunizers and consumers express more concern than other stakeholders regarding this impending change (Fig. 3), but there is a noticeable absence of proactivity. There is also currently no overriding policy body or cohesive national immunization plan in the U.S. to address these challenges.

The adult vaccine market has also seen a shift in focus to newer participants, such as the rise of alternative vaccination sites like pharmacies, aiding a decades long trend which is expected to continue. Historically, adult vaccines have been distributed through various sources like primary care offices, pharmacies, and vaccine clinics^24,25^. However, primary care doctors tend to prioritize pediatric immunization^26^. Surprisingly, 66.3% of health care professionals (HCPs) in the U.S. do not check their patients’ vaccine statuses with every visit, and more than half (53.0%) do not even include vaccine administration within their practice scope^27^. This could limit immunization access for patients who rely solely on their primary care physician. In recent years, pharmacists have emerged as popular alternative vaccinators for adults, offering convenience with longer operating hours and proximity^28–31^.

However, this shift brings with it its own set of challenges. Most physicians recognize the benefits and convenience of pharmacists sharing the role of vaccinating adults; however, concerns exist about pharmacists’ access to patient medical records and vaccination history^32^. In fact, a degree of discord often exists between physicians and pharmacists, particularly concerning communication and collaboration within the office environment. While a synergistic relationship between these two parties can significantly enhance patient care, physicians are typically hesitant to grant pharmacists the authority to administer vaccines or offer advice concerning medications to ‘their patients’^33^. On the other hand, pharmacists believe they possess the necessary skills to provide this support to patients, although they understand that many patients prefer receiving these recommendations directly from their physician.

Addressing these complexities requires fostering a sense of trust between pharmacists and physicians, which is paramount to the successful dissemination of future vaccines. It is crucial to nurture a “my pharmacist” mindset, where patients form a trusted bond with their pharmacist^33^. Regular interaction and cooperation between physicians and pharmacists are vital components of this success formula. Such collaboration allows the pharmacist to gain a comprehensive understanding of the patient’s medical history, thereby enabling them to identify any gaps in vaccination history. Furthermore, the establishment of a network comprising pharmacists collaborating closely with primary care physicians can bolster confidence in the pharmacists’ capability to administer vaccinations.

Another change brought on by the COVID-19 vaccine rollout is a feeling among many stakeholders, particularly GPs/PCPs and pharmacists, that they are prepared for the evolving adult vaccine landscape (Fig. 3). Some stakeholders during interviews drew parallels with the recent success of the COVID-19 vaccine distribution, expressing optimism that the expedited timelines witnessed during the pandemic can be replicated for future approved vaccines. This understanding underscores the confidence in our healthcare system’s resilience and adaptability, brought about by the unprecedented feats achieved during the pandemic. However, this view ignores the challenges faced during the COVID-19 vaccine rollout, which was initially slow and inefficient due to limited supply and logistical challenges^34^. The introduction of drive-through sites and mobile clinics increased access and speed^35^, but it is unlikely future vaccine rollouts will mirror this approach due to differences in urgency and resource allocation.

Stakeholders also anticipate that, in a non-pandemic environment, new offerings will be introduced gradually and cater to specific sub-populations (i.e., segmented recommendations), rather than blanket applications across the entire adult patient pool. However, it is crucial to remember that the post-COVID vaccine landscape is already witnessing the approval of new and innovative vaccines, such as vaccines for RSV, a trend which will likely continue over the next several years^36^. These products are expected to either target rare diseases, penetrate markets already served by existing vaccinations, and/or leverage the latest breakthroughs in vaccine technology. Such a dynamic environment calls for continued vigilance, flexibility, and a readiness to embrace change in our approach to vaccine administration and public health strategy.

Despite the expected increase in vaccine offerings, recommenders, funders, stockers/purchasers, and, to a lesser extent, immunizers anticipated limited challenges in adopting an expanded vaccine schedule. The lack of concern observed around the adoption of the expanded schedule likely mirrors a fragmented understanding among stakeholders. Each party tends to focus on its portion of the process, often overlooking the system’s holistic view and thereby underestimating the scale of change implicated. The adoption of expanding adult vaccines will be challenging in light of a lack of an existing cohesive national adult immunization schedule in the U.S. What currently exists is a category of approved products with recommendations governing their individual/disease area usage, which unfortunately may result in fragmented and inconsistent usage of adult vaccinations beyond what has already been established in the market (e.g., Influenza, Pneumo, Shingles).

### Lack of market standardization

While there was little concern in adopting an expanded vaccine schedule, stakeholders in every category acknowledged the challenge in strategically prioritizing adult vaccines. However, no individual stakeholder group is willing to take responsibility for establishing priorities or developing schedules.

Currently, the task of assessing the potential influence of vaccines on public health and establishing vaccination guidelines falls to Vaccine Technical Committees (VTCs), such as the ACIP in the Centers for Disease Control and Prevention (CDC), and the National Vaccine Advisory Committee (NVAC). ACIP plays a pivotal role as the primary policymaker in adult immunization. It not only influences reimbursement decisions but also provides a product-specific adult immunization schedule that outlines the required doses or boosters, taking into account the patient’s age and risk factors^37–40^. The recommendations made by the ACIP are reviewed and usually adopted by the CDC director^41^. Once adopted, these recommendations become official CDC guidelines and are published in the Morbidity and Mortality Weekly Report (MMWR).

When a vaccine is not recommended for routine use, the ACIP will issue a SCDM recommendation. Unlike routine, catch-up, and risk-based recommendations, SCDM vaccinations are not recommended for everyone in a particular age group or everyone in an identifiable risk group. Rather, SCDM recommendations are individually based and informed by a decision process between the health care provider and the patient or parent/guardian^42^, a process that encourages informed and collaborative discussions that guide mutual decisions about the suitability of a particular vaccine^43^, such as the newly approved RSV vaccines that are recommended, using SCDM, for adults 60 years and older^44^. Although HCPs have mixed responses to SCDM recommendations, the majority support them for certain vaccines^45^. However, concerns persist about the additional time required for patient discussions, potential confusion, and the need for specific talking points to guide these conversations.

An integrated vaccine tracking system, like a national immunization information system (IIS), could significantly ease the intricacies associated with adult vaccination delivery^46^. An IIS can serve as a one-stop repository for immunization records, thereby streamlining the process of vaccination tracking and ensuring accurate records. Regrettably, the U.S. does not currently have such a unified national system in place. Instead, the responsibility falls on each individual state to devise its own protocols and systems. State policies can range from opt-in only (Texas) to mandatory (New York), and one state, New Hampshire, does not have an IIS^47^. Without a centralized national vaccination tracking system, patients find themselves coordinating with their HCPs to trace their vaccine history. This often leads to incomplete or inaccurate records due to miscommunication, gaps in data transfer, or simple human error^47^.

The lack of a national immunization plan and national IISs imposes a significant burden on immunizers, who are required to rapidly assess the disease risk level for each patient and determine which vaccines are most critical to recommend during that visit and which can be deferred to a later date. This confusion, in conjunction with perceived inadequate training, time constraints, and lack of emphasis on vaccinations, can hinder HCPs’ ability to effectively administer vaccines^27^, leading to suboptimal patient health outcomes.

Despite the challenges brought about by lack of market standardization in the adult vaccine market, there is little momentum for change. Unlike the pediatric market, the adult vaccine market has not had the necessity to standardize, and it lacks the uniformity of the pediatric market that allowed for streamlined standardization of recommendations and schedules. Adult populations also have more choice as to whether they receive immunizations, with many younger adults believing that there is no real need to vaccinate until they are older.

Unfortunately, our research revealed that it is not clear which stakeholder group would drive an effort for standardization, and there is no clear distinction as to who would make recommendations for new adult vaccines and based on what factors. This is a critical gap as trade-offs for new vaccines must be assessed between different disease risk profiles and budgets, with some vaccines slated to receive funding while others will not.

### Estimating vaccine administration limits—‘The Battle of the Arm’

To quantify how patients and immunizers will navigate the increasing number of vaccine options, we assessed their perceptions on current and future vaccination habits. Specifically, we wanted to understand how many vaccines adults were willing to receive in a year and in a single appointment, and then compare that with the number of vaccination visits they intend to make each year.

**Fig. 4:**
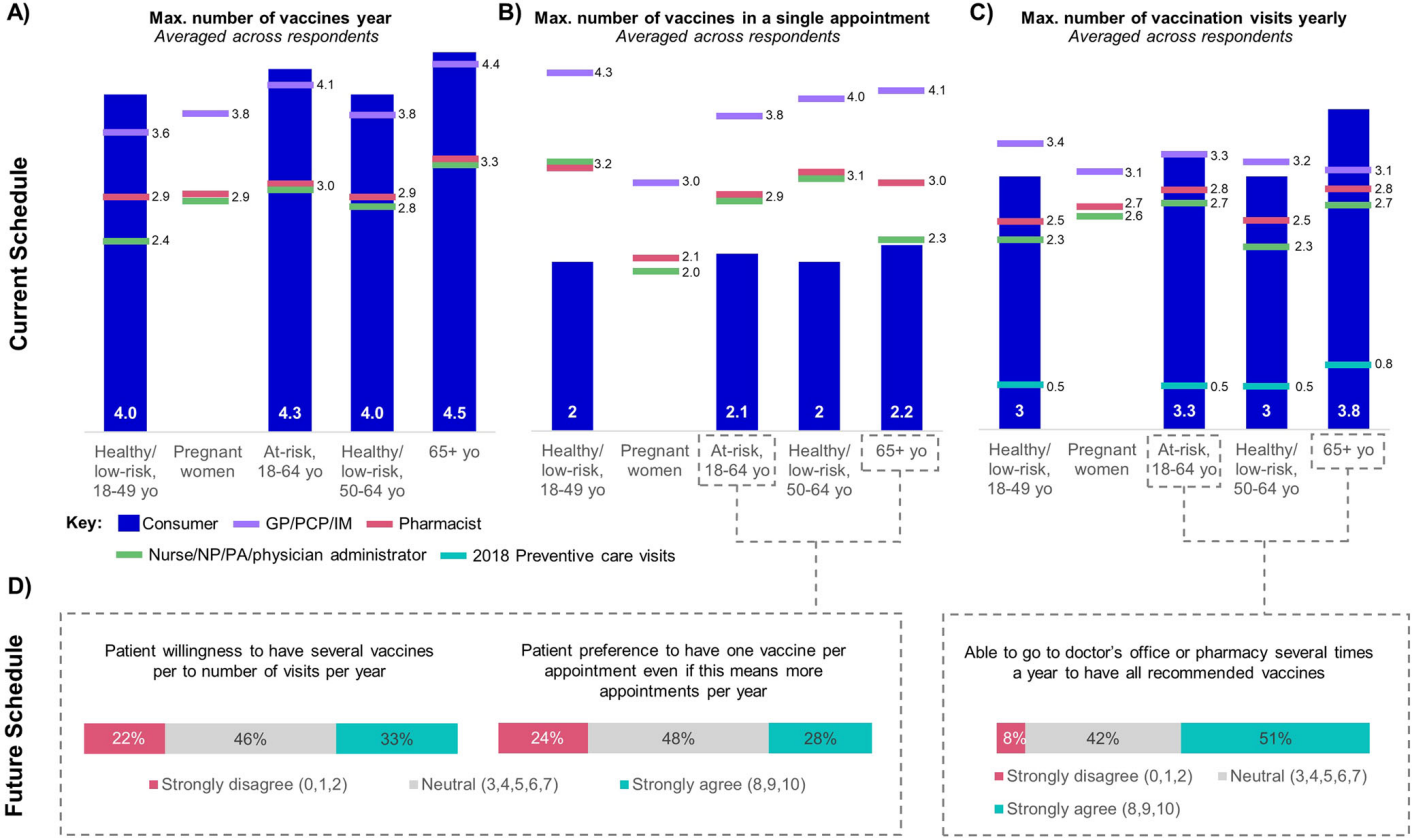
The battle for the arm as an expanded vaccine schedule drives complexity. A quantitative online survey conducted in the U.S. was used to derive consumer (*n* = 500) and immunizer (*n* = 103) perceptions regarding a crowded vaccine schedule. **A** Consumer and immunizer reports for the maximum number of vaccines patients are willing to receive yearly. Consumers were asked “what is the maximum number of different vaccines you would be willing to receive in a year (some possibly requiring several appointments)?” Immunizers were asked “what is the maximum number of vaccines you expect an individual adult patient would be willing to receive in a year?” Results presented are for a “typical” patient with regard to vaccine attitudes, or one who is “busy” (e.g., working full-time). **B** Immunizer reports for the maximum number of vaccines they would administer in a single appointment, and consumer reports for the maximum number of vaccines patients are willing to receive in a single appointment. Consumers were asked “what is the maximum number of vaccines (individual shots) you would be willing to receive in a single appointment?” Immunizers were asked “What is the maximum number of vaccines (individual shots) you would be willing to give a typical patient in a single appointment? Please assume co-administration is supported by relevant data.” **C** The reported number of vaccination appointments patients schedule as reported by patients and immunizers compared to actual visits indicated by red lines. Consumers were asked “What is the maximum number of visits for a vaccination you would be willing to attend per year?” Immunizers were asked “How many visits per year for the purposes of getting a vaccine do you think patients would be willing to attend per year (on average)?” Results presented are for a “typical” patient. 2018 data was obtained from the CDC NCHS Data Brief No. 408, May 2021. Consumer data was collected for healthy/low-risk 18–64-year-olds. This data is used for both groups - healthy/low-risk 18–49-year-olds and healthy/low-risk 50–64-year-olds. Consumer data was not collected for pregnant women. **D** Consumer attitudes within future vaccination schedules. Consumers were asked “Thinking about this future with many different vaccine options available for adults like you, to what extent do you agree with the following statements?”.

Patients across all age and risk groups, when surveyed, reported their willingness to receive up to four vaccines per year, a perception that aligns with that of the immunizers (Fig. 4A). Immunizers also appeared to overestimate the maximum number of vaccinations a patient would be willing to receive in a single appointment (Fig. 4B). When asked, all patient groups responded that they prefer to limit the number of shots to two per visit, one for each arm. This would necessitate multiple appointments throughout the year to reach the 4 immunizations patients are willing to receive each year. However, all stakeholders appear to overestimate the maximum number of yearly vaccination visits a patient is likely to make, with patients and immunizers estimating at least two appointments annually (Fig. 4B). This contradicts data from 2018 demonstrating that, on average, patients across all age groups made less than one preventative care appointment per year with their physician.

Consumers were also presented with the future vaccine schedule to gauge their willingness to adhere to future recommendations (Fig. 4D). Consumers (33%) still indicated a willingness to receive multiple vaccines per visit; however, 28% stated a strong preference to schedule more vaccination appointments rather than have more than 1 vaccine per visit. When asked if they would be able to attend more than one vaccination appointment per year in order to receive all recommended vaccines, 51% of patients strongly agreed. However, if the vaccination behavior observed in 2018 continues over the next decade, patients will need to be willing to receive vaccines at ‘non-wellness’ visits in order to receive the recommended number of vaccines.

The number of vaccination opportunities, however, is on the decline and the timeline within which to administer vaccines may become compressed. COVID-19 has increased the prevalence of telemedicine—acquiring medical care digitally - which may reduce face-to-face encounters between consumers and HCPs^48^. HCPs also must contend with forced seasonality, where nonseasonal vaccines are aligned or coadministered with flu vaccines. At this time, limited vaccination opportunities and forced seasonality do not represent significant barriers to immunization due to the manageable number of currently available vaccine products. Most of these products are also not seasonal and have primary series that do not have to be administered annually. However, as the number of vaccine products, particularly seasonal vaccine products, increase over the next couple of decades, HCPs will be faced with more immunizations, but still only one shot per arm per patient. This limited arm space will force the adult vaccine market to become embroiled in the ‘battle of the arm.’

To date, the limited variety of adult vaccines—just seven recommended by the CDC—has made managing vaccination schedules relatively straightforward (Supplementary Fig. 2). This has fostered a ‘there’s always next year’ attitude among patients. However, the increasing number of vaccines threatens to disrupt this status quo. Currently, adults aged 50 and 65 years old could be advised to receive a maximum of 2.8 and 3.5 of recommended vaccines, respectively, in a single visit depending on risk factors. These estimates were based on 50- or 65-year-old Americans (assuming average lifespan) who require all adult vaccinations, and who only received vaccines at annual appointments. In 10 years, we estimate that the number of recommended doses for adults aged 50 and 65 years is expected to increase to 5.6 and 6.4 per year, respectively.

The expansion of the adult vaccine market means that consumers will need to take more ownership of their vaccination schedules and records. Current vaccination policies are often developed around the assumption that consumer stakeholders will make rational decisions towards the betterment of their own health. However, behavioral economics and choice theory suggest that humans deviate from rational behavior in predictable patterns, especially when faced with an overwhelming number of options—a phenomenon known as choice paralysis. Unfortunately, our study revealed that an estimated 38% of patients already feel overwhelmed at the prospect of an increased volume of adult vaccines.

The COM-B (capability, opportunity, and motivation - behavior) model of behavior change, which suggests that vaccine uptake relies on capability, opportunity, and motivation, offers insights into strategies to overcome consumer choice paralysis and increase vaccine uptake^49^. As the number of available vaccines increases, HCPs will play a crucial role in helping patients navigate this changing landscape, making complex decisions about the prioritization and administration of vaccines. A physician’s recommendation, which has been proven to increase immunization rates^50^, can increase consumer’s knowledge (a form of capability) of available vaccines and provide persuasion (motivation) through the trust they have built with their patients. However, due to the limitations discussed above, physicians and other vaccine administrators may not have the capability, opportunity, or motivation to recommend immunizations to their patients. When interviewed, HCP respondents reported that they rely on morbidity and mortality weekly report (MMWR) recommendations to guide their vaccination choices and have no policy or training (planned or completed) to guide prioritization decisions and will vaccinate on patient preference. When shown the stimuli for the expected number of adult vaccines (Supplementary Fig. 4), they expressed a need for overarching recommendations on vaccine priority to be issued by the ACIP but doubt this will happen due to the complexities of adult care.

Delivering more vaccines via pharmacies and other non-traditional venues is one proposed solution for administering an increasing number of vaccines, though it calls for the creation of advanced data systems to ensure meticulous tracking of each patient’s vaccinations. Combination vaccines, which combine multiple vaccines into a single dose, could also help reduce the burden of prioritization and increase vaccine uptake. Though only one such vaccine currently exists for adults (Tdap for tetanus, diphtheria, and pertussis), more are anticipated in the future, including an influenza + COVID combination as well as a possible influenza + RSV combination.

The success of these solutions, however, hinges on patient acceptance. With vaccine hesitancy on the rise, patient acceptance is expected to be a major barrier to adult vaccination. In fact, the WHO has recognized vaccine hesitancy as one of the top ten global health threats^51^, and patient skepticism of new vaccines will likely increase if they are designed with new vaccine platforms and/or combinations. Therefore, education campaigns with trusted public health figures and community leaders will be key to improving patient acceptance of these vaccines. We must also look towards addressing the ‘Opportunity’ aspect of the COM-B model, as inequity in the adult vaccine landscape may limit access of new vaccines among disadvantaged demographics.

### Lack of incentives and accountability—equity falling through cracks

Despite the fact that attaining optimal health requires an acknowledgement of and targeted efforts to address social or societal conditions contributing to health disparities, far too many in the U.S. still suffer from unequal access to healthcare^52^. Nearly four in ten lower-income adults have reported delaying medical care due to cost^53^. Such disparities have caused a divide in actual health outcomes, with lower socioeconomic status leading to a shorter life expectancy^54^. Bridging the equity gap, however, is not just a moral imperative but also a financial one. In fact, estimates suggest that by eliminating these racial disparities alone, we could save over $90 billion annually in unnecessary medical expenses^55^.

The U.S.’ vaccination landscape has long been affected by existing health disparities, with lower rates of coverage among certain racial and socioeconomic groups. This has been historically true for influenza and pneumococcal vaccinations, particularly for vaccinations with newer technologies^56^. One study found that only approximately 13% of black influenza vaccine recipients received the newer high-dose inactivated trivalent influenza vaccine, compared to approximately 27% of white recipients^56^. The COVID-19 pandemic only exacerbated this gap, as attitude towards the vaccine further decreased uptake of the influenza vaccination^57^. Furthermore, data has revealed disparities in COVID-19 vaccination rates among various demographics such as race, ethnicity, household income, urbanicity, political affiliation, and others^58–61^. These disparities exist despite unprecedented efforts to make vaccination available, free of charge regardless of immigration or insurance status, and to make vaccination convenient^62,63^. Other interventions, such as the Affordable Care Act and Inflation Reduction Act have improved adult vaccine coverage for insured individuals^64,65^; however, they do not sufficiently support uninsured individuals or cover vaccines not recommended by the ACIP. Without targeted intervention, the introduction of new adult vaccines may worsen health equity gaps in the future.

Unfortunately, our market research has highlighted that no stakeholder group is individually accountable for addressing vaccine equity. We found a significant gap in understanding and responsibility within the vaccine ecosystem, leading to a disconcerting reality: equity is falling through the cracks. Each stakeholder we engaged was primarily focused on their specific goals, often assuming that someone upstream was responsible for ensuring equitable access to vaccines. This mindset is heavily influenced by cognitive biases such as groupthink - a psychological phenomenon where individuals seek conformity in a group, often ignoring contrary information or viewpoints, and anchoring bias—the tendency to heavily rely on the first piece of information encountered (the “anchor”) when making decisions^66,67^. Such biases create an environment where critical aspects of vaccine distribution and access are overlooked.

The most striking finding was the widespread belief that the ACIP shouldered the responsibility for implementing measures to ensure equity. However, this belief represents a fundamental misunderstanding of the ACIP’s role as an advisory committee that is responsible for creating and maintaining recommendations which consider a spectrum of equity measures, such as equitable inclusion in trials^68^. When interviewed, ex-ACIP members stated that they had no ability or remit to enforce activities beyond making recommendations.

This misplaced trust reverberates throughout the system, creating a perpetuating cycle where stakeholders’ focus on narrow objectives leaves broader equity issues unaddressed. The result is a systemic failure where lives are impacted or even lost due to diseases that could have been prevented with vaccination. For example, influenza hospitalization rates in the U.S. were nearly 80% higher among Black adults than White adults from 2009 to 2022^69^.

At the heart of this issue is the absence of systems thinking, or a holistic approach that takes into account structures, patterns of interaction, events, and organizational dynamics^70^. Stakeholders need to comprehend the interconnectedness and interdependencies within the vaccine ecosystem. Ensuring equity in vaccine access is a collective responsibility that requires the active involvement of all stakeholders, rather than being the sole obligation of a single entity.

Social cognition—a person’s ability to understand and function in the social world—plays a significant role in this context^71^. Cultural cognition, a subset of social cognition, influences how individuals perceive information and make decisions based on their cultural affiliations and identities^72^. For instance, a community’s collective understanding about vaccines can create a powerful social norm, influencing individuals’ attitudes and behaviors towards vaccination.

Behavioral economics provides valuable insights into these dynamics and how they can be addressed. For example, interventions that emphasize the social norm of vaccine acceptance can counteract the effects of groupthink by challenging the group’s consensus^71^. Likewise, understanding anchoring bias can guide the design of communication strategies. By ensuring the first piece of information individuals receive about vaccines is accurate and positive, healthcare providers can set a positive anchor that shapes subsequent perceptions^67^. However, these insights also highlight why many interventions to combat inequity achieve only limited or short-term success. They often fail to address the root causes of these biases and the systemic factors that contribute to them.

The most meaningful successes, such as the one seen at UT Southwestern, are comprehensive grassroots efforts that fill the void created by the current system^73^. These initiatives understand and address the local cultural cognition and social norms, leading to the effective promotion of vaccine acceptance within their target communities. However, the challenge of applying lessons learned from these grassroots initiatives lies in scaling these strategies nationally. Every community has its own cultural and social norms, meaning that interventions must be tailored and localized. Such efforts, which are often underfunded, require substantial community involvement and are highly resource-intensive, making them difficult, if not impossible, to scale.

While our analysis above has highlighted behavioral drivers that contribute to the issue of vaccine inequity, it is important to acknowledge that these factors are just one part of the larger systemic problem. Addressing behavioral drivers can help to mitigate some aspects of inequity, but it does not fully resolve the issue. In fact, a hyper-focus on factors behind vaccine hesitancy can often mask systemic issues, such as structural racism, that impact vaccine equity^74^. Even if we were successful in eliminating vaccine hesitancy, we would still face significant barriers in terms of access and affordability, particularly for lower-income and uninsured individuals^53,58^. This reality amplifies the need for a more comprehensive approach to vaccine equity beyond simply addressing vaccine hesitancy.

Moreover, the current reliance on downstream interventions, such as improving public awareness and promoting vaccine acceptance, often overlooks the upstream, structural issues that contribute to health disparities. To truly advance vaccine equity, we must adopt a holistic, end-to-end approach that transcends sectors and addresses all dimensions of the issue, including policy guidance, access, affordability, and social determinants of health. This includes forming multisectoral partnerships, focusing resources on the most vulnerable populations, and designing interventions that consider local dynamics. We need to prioritize equity in our monitoring and evaluation efforts, measuring not just outcomes but also impact. Only by doing so can we hope to ensure that equity does not fall through the cracks but forms the cornerstone of our healthcare system.

## Conclusion

The adult vaccine market stands on the threshold of significant growth in the forthcoming years. Preparing to incorporate more vaccines into the existing ecosystem could lead to enhanced health and economic outcomes in the future. The time is ripe for proactive solutions that consider the pivotal role of the consumer and their choices, along with improved coordination and accessibility in a currently fragmented landscape. Such measures encompass the elimination of barriers to vaccine access, streamlining processes for reimbursement and operations, enhancing record-keeping, and equipping immunizers and patients with the necessary tools to instill confidence in current and future vaccines. It is crucial to overcome these equity barriers before the surge in vaccine products leads to wider disparities. Innovation across policymakers, payers, and healthcare systems, including centralized digital records and policy driven by behavioral economics, will propel these solutions forward. As a result, adult vaccination coverage could mirror the successes seen within the pediatric vaccine ecosystem, ultimately positioning adult vaccination as a formidable shield against numerous life-threatening diseases.

### Supplementary information

Supplemental Information
