# Supplementary material for: Exploring the future adult vaccine landscape—crowded schedules and new dynamics
Source: NPJ Vaccines. 2024 Feb 9;9:27. doi: 10.1038/s41541-024-00809-z (PMC10858163; doi:10.1038/s41541-024-00809-z)
Supplement: Supplementary file 1 — Supplemental Information [file 41541_2024_809_MOESM1_ESM.pdf]

Supplementary Information

Supplementary Methods

Market Research Study Methodology

Study Design

Our research methodology was built on an iterative, sequential primary market research design using mixed methods. This encompassed a five-step process that began with fact gathering and hypothesis identification. The initial fact-gathering stage involved an extensive exploration of existing knowledge, data, and available information on the potential evolution of the adult vaccine landscape. This investigation covered publications, clinical trials, and product pipelines to establish a solid foundation for the research. Hypotheses on potential future market state scenarios were developed based on the results of the fact-finding stage. The study then proceeded with targeted qualitative interviews with key stakeholders across the value chain, designed to refine U.S. hypotheses, followed by quantitative surveys with U.S. Vaccine Purchasers, Immunizers and Consumers designed to characterize degree of impact and tradeoffs for U.S. future state scenarios.

Participants and Recruitment

For the qualitative interviews, we engaged with 53 U.S. stakeholders who filled various roles within the value chain. These included former ACIP members and policy makers, payers, self-insured employers, key opinion leaders (KOLs) who do not immunize, retail pharmacy purchasers, integrated delivery network (IDN) purchasers, group purchasing organizations (GPOs), wholesaler/distributors, immunizing healthcare providers (HCPs) including pharmacists, primary care physicians (PCPs), physician assistants (PAs), and patient advocacy groups (PAGs).

In addition, the study involved 683 stakeholders who participated in the quantitative survey. These were composed of stocking & purchasing stakeholders (n=80), immunizers (n=103), and consumers (n=500) selected based on specific demographic and health criteria.

Discussion Guide

During the qualitative interviews, we presented various stimuli to the participants to facilitate conversation. These included showcards that illustrated the evolution of the adult vaccine landscape across disease states over time, highlighted current and future disease targets and vaccines, and depicted the current and potential future ACIP recommendations. Additionally, we presented two future scenarios for the development of the adult vaccines market. The first scenario was optimistic, showing a future where stakeholders adapt and seize new opportunities, while the second scenario was pessimistic, illustrating a future where stakeholders maintain their usual practices that might not fit the evolving market.

Methodology for determining projected future vaccine volume expansion in the United States
(Fig. 3B)

The methodology for determining the projected vaccine dose volume through 2032, categorized by vaccine type, involved several steps. Vaccine types assessed were Seasonal, One-time,

Routine, and Additional Seasonal. Indications within these types included Influenza (18-49, 50-64, 65+), COVID (18-49, 50-64, 65+), RSV (Pregnant, 65+), Pneumococcal, Hep-B, Zoster, Meningococcal, HPV, HIV, CMV, Tdap, Hep-A, HMPV, and PIV.

First, current eligible populations, by disease indication, were determined using U.S. census data from 2021. A US population compound annual growth rate (CAGR) of 1.6% was applied to estimate the US population in 2023. Populations were segmented by age group for each disease indication, which were determined using the ACIP recommendations. For indications where recommendations for at-risk populations existed, at-risk-rates were determined (**Supplementary Table 1**) and total at-risk populations were included within the eligible population. Projected eligible populations from 2024 through 2032 were calculated assuming a CAGR of 1.6%.

Supplementary Table 1. At-Risk rates and population statistics and assumptions.

**At-Risk Rates**		
Immunocompromised	3%	High-Risk Levels
Pregnancy	3%	% all people 15-44 - CDC
MSM	2.5%	Of total population
SCDM	10%	Assumption-based
Travel: At-Risk	13%	Pre-COVID International Travel Rate
Hep: At-Risk	8%	MSM + Healthcare Professionals

Current coverage rates for each vaccine indication were determined and are shown in **Supplementary Table 2**.

Supplementary Table 2. Current Coverage Rates

Vaccine Type	Indication	Current Coverage Rate	Groups Covered	Source
Seasonal	Influenza: 18-49	38%	18-49	1
Seasonal	Influenza: 50-64	54%	50-64	1
Seasonal	Influenza: 65+	75%	65+	1
Seasonal	COVID: 18-49	20%	18-49	2
Seasonal	COVID: 50-64	32%	50-64	2
Seasonal	COVID: 65+	45%	65+	2
Seasonal	RSV: Pregnant	25%	Pregnant	Assumption
Seasonal	RSV: 65+	25%	65+	Assumption
One-Time	Pneumococcal	82%	65+	2
One-Time	Hep-B	30%	All Adults	3
One-Time	Zoster	39%	60+	4
One-Time	Meningococcal	54%	All Adults	5
One-Time	HPV	22%	18-26	6
One-Time	HIV	85%	MSM & Immunocompromised	Assumption: High Interest
One-Time	CMV	50%	Pregnant & Immunocompromised	Assumption: Similar to other routine vaccinations
Routine	Tdap	63%	All Adults	4
Routine	Hep-A	12%	All Adults	7

The 2032 coverage rate for each vaccine indication was estimated using the following assumptions:

- Influenza (18-49): Combo influence significant, original coverage rates included immunocompromised & high-risk (~20% of population). New innovation, large expenditure by major companies.
- Influenza (50-64): Growth potential identified.
- Influenza (65+): Little change expected due to high penetration.
- COVID (18-49, 50-64, 65+): Expected to mirror influenza patterns, combined public messaging considered.
- RSV (Pregnant, 65+): Identified as priority areas with significant competition & innovation.
- Pneumococcal: Stable but expected to overcome pandemic spillover effect by ten years - steady growth until then.
- Hep-B, Meningococcal, HPV: Minimal growth in coverage anticipated.
- Zoster: Room for further penetration with additional launches.
- CMV: Expected to follow similar trajectory to other routine vaccines.
- Tdap, Hep-A: No growth anticipated.

Coverage rates over time from years 2024 through 2031 (**Supplementary Table 3**) were calculated using the following equation:

$$Coverage\ Rate_x = \frac{(Coverage\ rate_{2032} - Coverage\ rate_{2023})}{(2032 - x)} + Coverage\ rate_{x-1},$$

where *x* = *year*.

Launch dates for RSV, HIV, CMV, HMPV, and PIV vaccines were set at 2025, 2028, 2025, 2028, and 2028, respectively, and were taken into account when determining what year coverage begins.

Supplementary Table 3. Coverage Rate over Time

Vaccine Type	Indication	2023	2024	2025	2026	2027	2028	2029	2030	2031	2032
Seasonal	Influenza: 18-49	38%	40%	42%	43%	45%	47%	49%	51%	53%	55%
Seasonal	Influenza: 50-64	54%	57%	59%	61%	63%	66%	68%	70%	73%	75%
Seasonal	Influenza: 65+	75%	76%	77%	78%	80%	81%	82%	83%	84%	85%
Seasonal	COVID: 18-49	20%	24%	27%	31%	35%	39%	43%	47%	51%	55%
Seasonal	COVID: 50-64	32%	37%	42%	47%	51%	56%	61%	66%	70%	75%
Seasonal	COVID: 65+	45%	50%	54%	59%	63%	67%	72%	76%	81%	85%
Seasonal	RSV: Pregnant	0%	0%	25%	30%	35%	40%	44%	46%	48%	50%
Seasonal	RSV: 65+	0%	0%	25%	32%	39%	46%	54%	61%	68%	75%
One-Time	Pneumococcal	82%	83%	84%	85%	86%	88%	89%	90%	91%	92%
One-Time	Hep-B	30%	31%	31%	32%	32%	33%	33%	34%	34%	35%
One-Time	Zoster	39%	41%	43%	44%	46%	48%	50%	51%	53%	55%
One-Time	Meningococcal	54%	55%	56%	57%	58%	60%	61%	62%	63%	64%
One-Time	HPV	10%	10%	10%	10%	10%	10%	10%	10%	10%	10%

Vaccine Type	Indication	2023	2024	2025	2026	2027	2028	2029	2030	2031	2032
One-Time	HIV	0%	0%	0%	0%	0%	80%	82%	85%	87%	90%
One-Time	CMV	0%	0%	25%	27%	30%	32%	35%	38%	39%	40%
Routine	Tdap	63%	63%	63%	63%	63%	63%	63%	63%	63%	63%
Routine	Hep-A	12%	12%	12%	12%	12%	12%	12%	12%	12%	12%
**Additional Seasonal Vaccines**											
Seasonal	HMPV	0%	0%	0%	0%	0%	25%	33%	40%	48%	55%
Seasonal	PIV	0%	0%	0%	0%	0%	25%	33%	40%	48%	55%

Newly covered populations per year for each vaccine indication was determined by multiplying the eligible population per year by the coverage rate.

The total dose volume per year (**Supplementary Table 4**) was calculated for each vaccine indication by multiplying the newly covered populations per year by the number of doses in each vaccine's regimen. This dose number was determined using the ACIP recommendations.

Supplementary Table 4. Covered Doses per Year

Vaccine Type	Indication	2023	2024	2025	2026	2027
Seasonal	Influenza: 18-49	47,335,206	50,544,681	53,844,741	57,237,464	60,724,970
Seasonal	Influenza: 50-64	32,264,256	34,178,258	36,145,248	38,166,433	40,243,042
Seasonal	Influenza: 65+	39,270,686	40,476,751	41,711,357	42,975,108	44,268,621
Seasonal	COVID: 18-49	24,630,038	30,039,392	35,615,539	41,362,433	47,284,110
Seasonal	COVID: 50-64	19,265,690	22,439,108	25,709,144	29,078,077	32,548,234
Seasonal	COVID: 65+	23,704,076	26,418,373	29,213,458	32,091,224	35,053,599
Seasonal	RSV: Pregnant	-	-	935,837	1,140,973	1,352,433
Seasonal	RSV: 65+	-	-	13,476,530	17,604,198	21,860,502
One-Time	Pneumococcal	7,743,859	7,974,370	8,210,275	8,451,687	8,698,723
One-Time	Hep-B	7,000,000	7,243,704	7,493,414	7,749,260	8,011,376
One-Time	Zoster	3,683,055	3,912,558	4,148,463	4,390,915	4,640,064
One-Time	Meningococcal	3,010,623	3,121,731	3,235,624	3,352,362	3,472,007
One-Time	HPV	1,425,266	1,448,070	1,471,239	1,494,779	1,518,695
One-Time	HIV	-	-	-	-	-
One-Time	CMV	-	-	58,797	64,517	72,833
Routine	Tdap	8,200,000	8,331,200	8,464,499	8,599,931	8,737,530
Routine	Hep-A	2,200,000	2,235,200	2,270,963	2,307,299	2,344,215
Seasonal	HMPV	-	-	-	-	-
Seasonal	PIV	-	-	-	-	-
**Total**		**219,732,754**	**238,363,395**	**272,005,129**	**296,066,660**	**320,830,954**

Vaccine Type	Indication	2028	2029	2030	2031	2032
Seasonal	Influenza: 18-49	64,309,423	67,993,033	71,778,055	75,666,792	79,661,593
Seasonal	Influenza: 50-64	42,376,332	44,567,585	46,818,110	49,129,242	51,502,345
Seasonal	Influenza: 65+	45,592,525	46,947,461	48,334,083	49,753,058	51,205,068
Seasonal	COVID: 18-49	53,384,692	59,668,388	66,139,495	72,802,403	79,661,593
Seasonal	COVID: 50-64	36,121,992	39,801,778	43,590,070	47,489,398	51,502,345
Seasonal	COVID: 65+	38,102,555	41,240,104	44,468,300	47,789,241	51,205,068
Seasonal	RSV: Pregnant	1,570,368	1,755,043	1,864,175	1,976,350	2,091,637
Seasonal	RSV: 65+	26,248,501	30,771,320	35,432,149	40,234,247	45,180,942
One-Time	Pneumococcal	8,951,500	9,210,139	9,474,763	9,745,498	10,022,470
One-Time	Hep-B	8,279,895	8,554,956	8,836,699	9,125,268	9,420,809
One-Time	Zoster	4,896,062	5,159,063	5,429,227	5,706,716	5,991,694
One-Time	Meningococcal	3,594,623	3,720,274	3,849,026	3,980,945	4,116,100
One-Time	HPV	1,542,995	1,567,683	1,592,765	1,618,250	1,644,142
One-Time	HIV	169,835	176,866	186,270	193,703	203,589
One-Time	CMV	78,931	87,712	96,754	100,889	105,132
Routine	Tdap	8,877,331	9,019,368	9,163,678	9,310,297	9,459,261
Routine	Hep-A	2,381,723	2,419,830	2,458,548	2,497,884	2,537,851
Seasonal	HMPV	14,133,808	18,667,934	23,343,533	28,163,973	33,132,691
Seasonal	PIV	14,133,808	18,667,934	23,343,533	28,163,973	33,132,691
**Total**		**374,746,898**	**409,996,470**	**446,199,234**	**483,448,126**	**521,777,019**

<u>Supplementary Figures</u>

Supplementary Figure 1. Size of the bubble indicates number of risk-adjusted assets in each phase of development, assuming all pipeline assets become commercially available, with Phase III assets in the short term (1-3 years), Phase II assets in the mid-term (3-5 years), and Phase I assets in the long term (5+ years). Size of bubble has been risk-adjusted using Pfizer's standard probability of technical and regulatory success (PRTS) values by stage of development (Exploratory Screen Development [ESD] - 60%; Screening Design Synthesis [SDS] - 45%; Lead Development [LD] - 55%; Preclinical - 55%; Phase I - 75%; Phase II - 70%; Phase III - 85%; Registration - 95%; Total PTRS = 3.5%). This is not exhaustive and excludes disease areas with fewer than five vaccines in pipeline, such as anthrax, hepatitis A, norovirus, typhoid, yellow fever, cholera, and Japanese encephalitis, except Lyme, Middle East Respiratory Syndrome (MERS), and klebsiella (nosocomial). Data were acquired from Informa Vaccines and Registered Vaccines query (March 2022) and manufacturer websites.

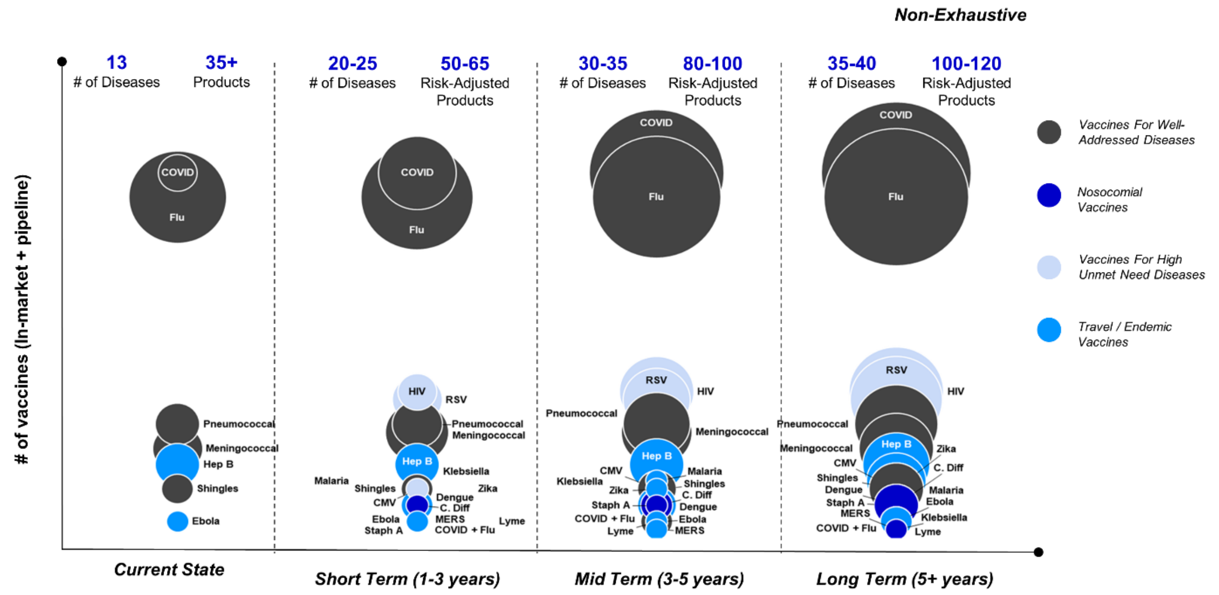

Supplementary Figure 2. Current and Future Adult Vaccine Schedules, by Disease Category

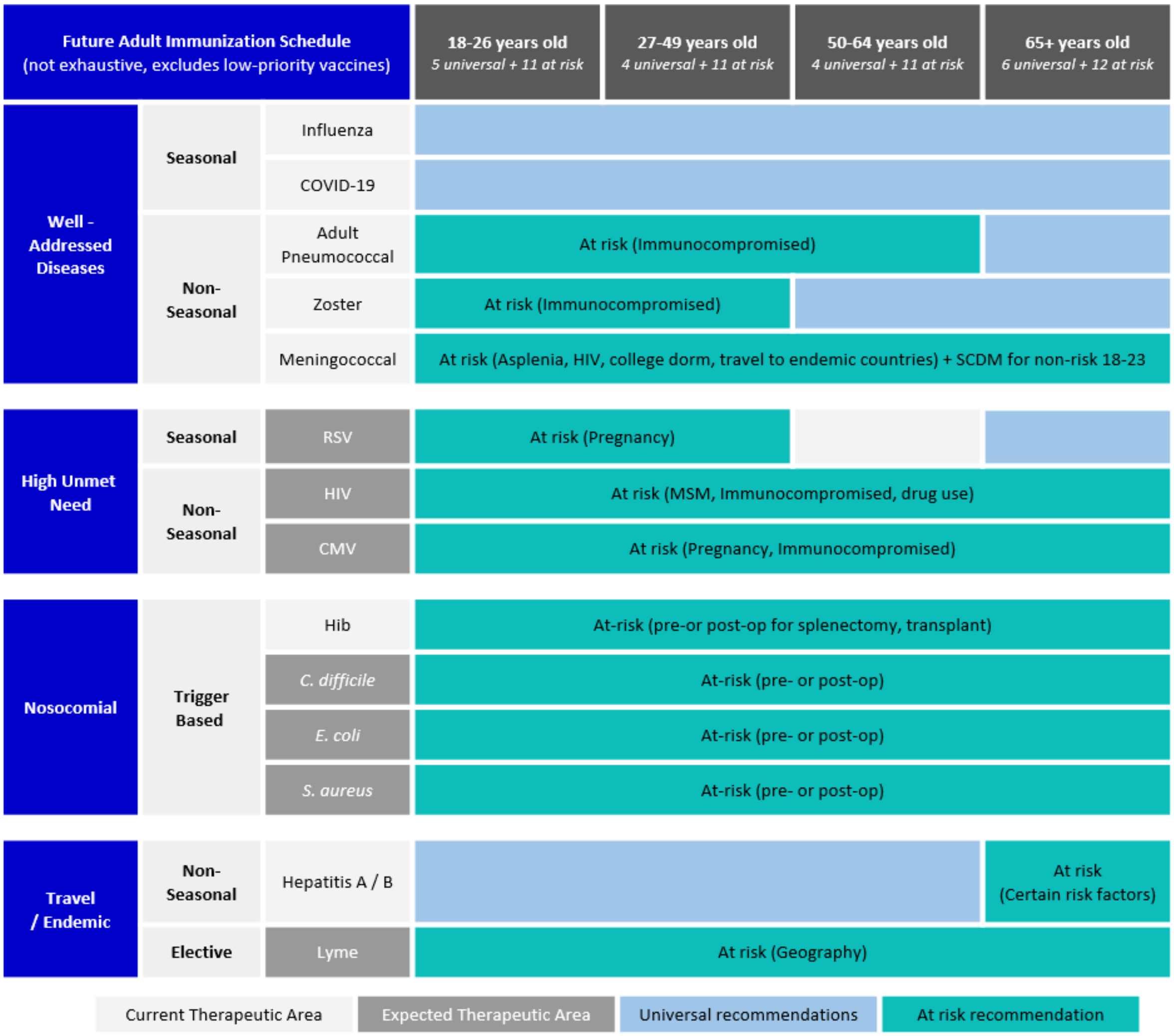

Supplementary Figure 3. Study Methodology

Qualitative Phase

**Methodology**	60 min web-assisted in-depth qualitative interviews

Value chain

Target	Stakeholder type	Stakeholder breakdown
**Recommenders & Funders**	ACIPs / Policymakers	**4**
	Payers	**6**
	Self-insured employers	**3**
	KOLs (non-immunizing)	**4**
**Stocking & Purchasing**	Retail Pharmacy Purchasers	**4**
	IDN Purchasers	**10**
	GPOs	**3**
	Wholesalers / Distributors (W/D)	**4**
**Immunizers/ Advocacy**	Immunizers HCPs (Pharmacists, PCPs, PAs)	**12**
	Patient Advocacy Groups	**3**

Quantitative Phase

**Methodology**	20 min online survey for Consumers / 30 min online survey for Stocking/Purchasing stakeholders and Immunizers

Target	Stakeholder type	Stakeholder breakdown
**Stocking & Purchasing**	Retail Pharmacy Purchasers	**50**
	IDN Purchasers	**30**
**Immunizers**	PCPs	**60**
	Nurse / NP / PA + Physician administrator	**23**
	Retail pharmacists	**20**
**Consumers***	65 + years old	**242**
	18-64 comorbid (has a risk condition recommended for vaccination from the CDC) +/- immunocompromised	**186**
	18-64 NOT comorbid (does NOT have a risk condition recommended for vaccination from the CDC) or immunocompromised	**72**

Supplementary Figure 4. Evolution of the adult vaccine landscape across disease states over time

ALL STAKEHOLDERS

Showcard 1: Evolution of the adult vaccine landscape across disease states over time

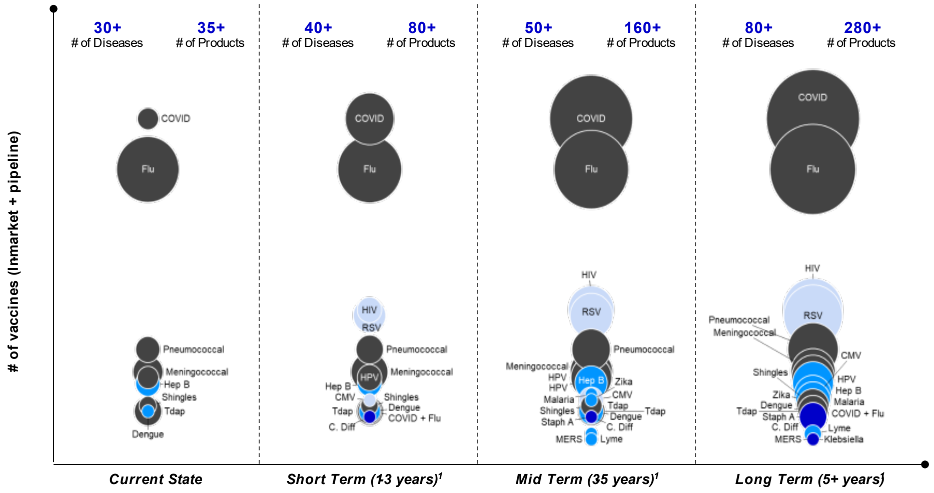

Size of the bubble indicates # of assets in each phase of development, [1]Assuming all pipeline assets become commercially available, phase III assets in the short term, phase II assets in the mid term, and phase I assets in the long term
the long term [2]Not exhaustive, excludes disease areas with less than 5 vaccines in pipeline such as anthrax, hep A, typhoid, yellow fever, japanese encephalitis, except lyme, MERS, and klebsiella (nosocomial) as they are Pfizer's area of interest
[2]Not exhaustive, excludes disease areas with less than 5 vaccines in pipeline such as anthrax, hep A, typhoid, yellow fever, japanese encephalitis, except lyme, MERS, and klebsiella (nosocomial) as they are Pfizer's area of interest
Source: Informa

Supplementary Figure 5. Showcard 2: Vaccine / disease categories

ALL STAKEHOLDERS

Showcard 2: Vaccine / disease categories

	Diseases	Vaccines
Current	~30	35+
Future (10 yrs)	~80	280+

Disease Category	**Well addressed diseases**<br>**Examples: Flu, COVID19, adult pneumococcal, HPV, Zoster, Tdap**<br>Prophylactic for prevalent diseases that are adequately addressed with approved or pipeline products	**High unmet need**<br>**Examples: RSV (maternal, elderly), CMV, HIV**<br>Prophylactic for global diseases with a high disease burden and large unmet need	**Nosocomial**<br>**Examples: C. difficile; S. Aureus**<br>Prophylactic for infections acquired in a hospital	**Travel/Endemic**<br>**Examples: Hepatitis A/B, ZIKA, Lyme, Rabies, Dengue**<br>Prophylactic for infections acquired in geographies for local populations and/or travelers
ACIP Reco.	Based on age segment and presence of risk factors	Based on age segment and presence of risk factors	Based on specific criteria e.g., eligible surgeries	Discretionary, restrictive recommendation
Strength	Recommendation, Conditional Recommendation, No Recommendation*			
Preference	Recommended for adults meeting age requirement, adults with additional risk factors/indications, based on shared clinical decision-making, and/or no recommendation			

* Based on HICPAC updates made to CDC recommendation categories

Hypothetical: For market research purposes only

Supplementary Figure 6. Illustrative adult immunization schedule

ACIP / POLICY, PAYERS / EMPLOYERS, ADVOCACY GROUPS, KOLS, IMMUNIZERS

Showcard 3: Illustrative adult immunization schedule - Version 1

	Diseases	Vaccines
Current	~30	35+
Future (5-10 yrs)	~80	280+

	Periodicity	Vaccine	Adult Age Group Target	Offerings
Well addressed diseases<br>*12 → 20+*	Seasonal	Flu	All Adult (18+)	Seasonal
		COVID-19	All Adult (18+)	Seasonal
	Routine (non-seasonal)	Meningococcal	All Adult (18+)	1 + 1
		Adult Pneumococcal	Older Adult (65+)<br>< 65 (if at-risk)	1 dose
		HPV	Adult < 50	3 dose series
		Zoster	Older Adult (50+)<br>< 50 (if immunocompromised)	2 dose series
		Tdap	All Adult (18+)<br>(+ each pregnancy, wound management)	1 + 1
High Unmet Need<br>*0 → 20+*	Seasonal	RSV	Maternal (women of childbearing potential), Older Adult	2 dose series
	Routine (non-seasonal)	HIV	All Adult (18+)	4 dose series
		CMV	All Adult (18+)	3 dose series
Nosocomial<br>*0 → 5+*	Trigger Based (pre-surgery, post-exposure)	C. difficile	All Adult (18+)<br>(with primary focus on 50+)	3 dose series
		S. aureus	Adult (18-65)*	1 dose
Travel/Endemic<br>*15+ → 35+*	Routine (non-seasonal)	Hepatitis A / B	All Adult (18+)	3 dose series
	Elective (pre-travel, post-exposure)	ZIKA	Adult (18-65)*	5 dose series
		Lyme	Adult (18-65)*	3 dose series + 1
		Rabies	All Adult (18+)	4 dose series
		Dengue	All Adult (18+)	3 dose series
		Tick-borne Encephalitis	All Adult (18+)	3 dose series
		Japanese Encephalitis	All Adult (18+)	2 dose series + 1

* Based specified age eligibility criteria in ongoing clinical trials

Hypothetical: For market research purposes only

KEY: Currently in market | New launch

Supplementary Figure 7. Consumer Showcard

CONSUMER SHOWCARD 1

- Over the next 5-10 years the number of vaccines for adults is expected to increase significantly meaning:
  - There will be **more options** for vaccine brand within existing vaccine categories such as flu, COVID, HPV
  - Vaccines covering **new diseases** for which there are no vaccines currently will be launched such as vaccines for HIV, Respiratory Syncytial Virus (RSV), Zika and others
- This is an example of what the adult vaccine schedule might look like in the future:
  - Diseases with current vaccines are in the blue rows – in the future there may be more options in these categories
  - Diseases for which there may be new vaccines coming on to the market in the next 5-10 years (but no current options exist) are in the yellow rows.

Type of vaccine	Vaccine
Seasonal	Flu
	COVID-19
	RSV (Respiratory Syncytial Virus)
Routine (non-seasonal)	Meningococcal
	Pneumococcal
	HPV (Human Papillomavirus)
	Zoster (Shingles)
	Tdap (Tetanus, diphtheria and pertussis (whooping cough))
	HIV (Human Immunodeficiency Virus)
	CMV (Cytomegalovirus)
For the prevention of hospital acquired infections, would be used before surgery or after exposure in certain groups of people	C. difficile
	S. aureus
Routine (non-seasonal)	Hepatitis A or B
Optional travel vaccines that could be used before travel or after exposure	ZIKA
	Lyme
	Rabies
	Dengue
	Tick-borne Encephalitis
	Japanese Encephalitis

Supplementary Figure 8. Show Card 5: Future scenarios for how the adult vaccines market may develop

Showcard 5: Future scenarios for how the adult vaccines market may develop

SCENARIO A … The future state market evolves and stakeholders are resilient and adapt their strategies to capitalize on new opportunities

- Adult immunization increases across all disease categories, similar to what is observed for US pediatrics flu coverage (~60 70%)
- Innovation is encouraged and there is strong demand from immunizers and patients
- High vaccination rates

SCENARIO B… The future state market evolves but stakeholders employ "business-as-usual" approach which does not fit the mold

- Adult immunization declines in some categories (<30%) and remains similar to current state or increases in other categories
- Innovation faces barriers and there is modest market demand
- Lower vaccination rates
- Tepid market uptake of some newer vaccine categories (nosocomial, travel/ endemic), though there is an improvement on current state

Hypothetical: For market research purposes only

Supplementary References

1 La, E. M. *et al.* Meningococcal B vaccination coverage among older adolescents in the United States. *Vaccine* **39**, 2660-2667 (2021).

2 WHO. *Pneumococcal vaccination coverage*, https://immunizationdata.who.int/pages/coverage/pcv.html?CODE=USA&ANTIGEN=PCV3&YEAR= (2023).

3 Weng, M. K. *et al.* Universal hepatitis B vaccination in adults aged 19–59 years: updated recommendations of the Advisory Committee on Immunization Practices—United States, 2022. *Am J Transplant* **22**, 1714-1720 (2022).

4 Control, C. f. D. & Prevention. Vaccination coverage among adults in the United States, national health interview survey, 2019-2020. *Centers for Disease Control and Prevention. Retrieved February* 27, 2023 (2022).

5 WHO. *Meningococcal vaccination coverage*, https://immunizationdata.who.int/pages/coverage/men.html?CODE=CAF&ANTIGEN=&YEAR= (2023).

6 WHO. *Human Papillomavirus (HPV) vaccination coverage*, https://immunizationdata.who.int/pages/coverage/hpv.html?CODE=AFR&ANTIGEN=&YEAR= (2023).

7 Nelson, N. P., Yankey, D., Singleton, J. A. & Elam-Evans, L. D. Hepatitis A vaccination coverage among adolescents (13-17 years) in the United States, 2008-2016. *Vaccine* **36**, 1650-1659 (2018).
